# Lipid profile in oral submucous fibrosis

**DOI:** 10.1186/1476-511X-8-29

**Published:** 2009-07-24

**Authors:** Ravi Mehrotra, Shruti Pandya, Ajay Kumar Chaudhary, Himanshu Pratap Singh, Ritesh Kumar Jaiswal, Mangal Singh, SC Gupta, Mamta Singh

**Affiliations:** 1 Department of Pathology, M.L.N Medical College, Allahabad, India; 2 Centre for Biotechnology, University of Allahabad, Allahabad, India; 3 Department of Otolaryngology, M.L.N Medical College, Allahabad, India

## Abstract

**Background:**

Changes in lipid profile have long been associated with malignancies as lipids play a key role in maintenance of cell integrity. This study evaluated the alterations in extended lipid profile in untreated patients of oral submucous fibrosis (OSMF) and studied the correlation between lipid levels with tobacco consumption.

**Materials and methods:**

In this hospital-based study, 65 clinically diagnosed and histopathologically proven patients of OSMF and 42 age and sex matched controls were studied. In these samples serum lipids including: (i) Total cholesterol, (ii) LDL cholesterol (LDLC), (iii) HDL cholesterol (HDLC) (iv) VLDL cholesterol (VLDLC) (v) triglycerides (vi) Apo-A1 (viii) Apo-B and (viii) LPa were analyzed.

**Results:**

A significant decrease in plasma total cholesterol, HDLC and Apo-A1 was observed in patients with OSMF as compared to the controls. Thus an inverse relationship between plasma lipid levels and patients was found in OSMF.

**Conclusion:**

The lower levels of plasma cholesterol and other lipid constituents in patients might be due to their increased utilization. The findings strongly warrant an in-depth study of alterations in plasma lipid profile in patients with oral precancerous conditions.

## Background

Oral submucous fibrosis (OSMF) is a chronic disease of the oral cavity, which is characterized by an epithelial and subepithelial inflammatory reaction followed by fibroelastic changes in the submucosa [1]. This disease occurs most commonly in South East Asia but cases have been reported worldwide in countries like Kenya, China, UK, Saudi Arabia and other parts of the world [2]. In India, the authors earlier reported that oral submucous fibrosis constituted the highest number of patients in premalignant group in a tertiary level hospital based study at Allahabad [3]. Tilakaratne et al. reported that areca nut is the main etiological factor for OSMF [4]. Pandya et al., again from Allahabad, confirmed that dohra/paan masala containing areca nut is a major risk factor of OSMF, especially in the younger age group. They also reported an increase in histopathological grading with severity and duration of addiction habit [5]. Excessive use of areca nut may cause fibrosis due to increased synthesis of collagen and induce the production of free radicals and reactive oxygen species, which are responsible for high rate of oxidation/peroxidation of polyunsaturated fatty acids which affect essential constituents of cell membrane and might be involved in tumorigenesis [6,7].

Lipids are major cell membrane components essential for various biological functions including cell growth and division of normal and malignant tissues. They are homogeneous group of compounds related more by physical than chemical properties. An alteration in the circulatory cholesterol levels has been found to be associated in the etiology of breast cancer and colorectal cancer [8]. However, only a few reports are available on plasma lipid profile in head and neck lesions [9,10].

A careful search of the literature does not reveal any research in extended lipid profiles in OSMF, and to the best of our knowledge this is the first report of its kind.

## Materials and methods

A hospital based study was conducted in 65 clinically diagnosed and histopathologically proven patients of OSMF attending the outpatient department in the division of Otolaryngology of the Swaroop Rani Nehru Hospital, a tertiary level referral hospital affiliated to the Moti Lal Nehru Medical College, Allahabad, India after the institutional ethical committee clearance. All the patients underwent recording of signs, symptoms, detailed history including habits, histopathology and extended lipid profile estimation. 42 healthy individuals, matched for age and sex, who had no complaint or any other major illness in recent past, were included as controls.

Fasting blood samples were collected in plain vials. Serum was collected after centrifugation and stored at -80°C until analyzed. Plasma levels of cholesterol, triglycerides, VLDL, HDL and LDL were calculated by using Autospan Reagents (Span Diagnostics, India). Simultaneously, Apo-A_1_, Apo-B and LPa were estimated by Electrophoresis by the standard technique. Statistical analysis, including calculation of coefficient of variation and Standard deviation was performed utilizing SPSS package. (Tokyo, Japan)

## Results

**Figure 1:**
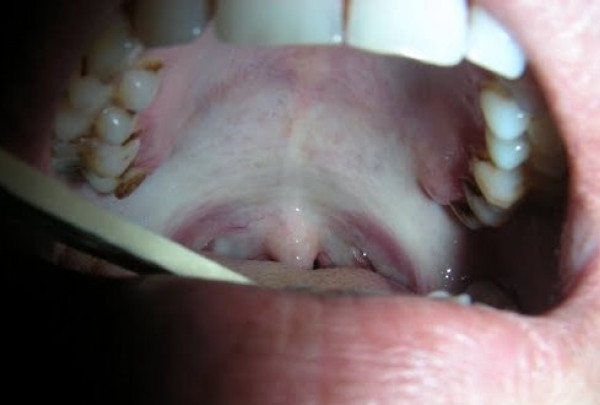
**Showing clinical picture (blanching and fibrosis) in oral submucous fibrosis**.

Sixty five cases of OSMF were studied. Maximum patients (30; 46.2%) were in their second decade (21–30 yrs) of life with a male predominance. According to the addiction habits, 26 (40%) consumed paan masala or dohra (a locally available combination of slaked lime and areca nut), 30.8% patients were addicted to betel quid with tobacco while 16 (24.6%) were smokers. Only 3 (6.6%) did not have any habit. 12 (18.5%) patients had a burning sensation in mouth, 16 (24.6%) had ulceration in the oral cavity, 15 (23%) had excessive salivation while 22 (33.7%) had difficulty in opening the mouth (trismus) (Figure 1). On clinical grading of trismus, 5 patients were found to have grade I (>3 cm), 34 had grade II (2–3 cm), and 26 had grade III (<2 cm) (Table 1). 28 patients consumed paan masala and betel quid with tobacco for 11–15 years (1–10 times/day), while 17 patients were addicted for 6–10 years (11–20 times/day) (Table 2).

**Table 1:** Clinico-pathological attributes in patients of OSMF

**Details**	**N = 65**	**(%)**
**Age (Years)**		

≤11–20	07	(10.8)

21–30	30	(46.2)

31–40	17	(26.2)

41–50	04	(6.1)

>51–60	07	(10.8)

**Gender**		

Male	59	(91)

Female	06	(9)

**Personal habits**		

Pan masala or Dohra	20	(30.8)

Betel quid with tobacco	16	(24.6)

Smoker (cigarettes/bidis)	26	(40)

No habit	03	(6.6)

**Clinical Symptoms**		

Burning sensation in mouth	12	(18.5)

Ulceration of oral cavity	16	(24.6)

Difficulty in mouth opening	22	(33.7)

Excessive salivation	15	(23.0)

**Clinical Signs**		

Grade I (>3 cm)	05	(7.7)
Grade II (2–3 cm)	34	(52.3)
GradeIII (< 2 cm)	26	(40)

**Table 2:** Distribution of tobacco consumption in relation to duration and no. of Pan Masala/Betel quids with tobacco/cigarettes/bidis smoked/day

**Duration in Year**	**Mean**	**No. of Pan Masala/Betel quid with tobacco/cigarettes/bidis smoked/day**		
		
		1–10 times/day	11–20 times/day	Total
1–5	03	04	06	10

6–10	08	08	09	17

11–15	13	18	10	28

16–20	18	06	04	10

**Figure 2:**
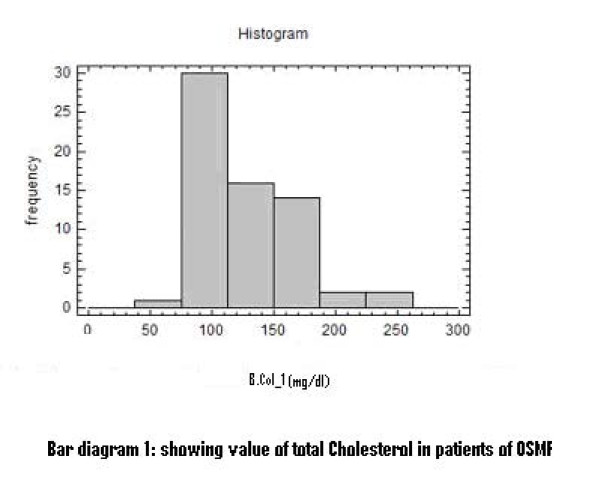
**Showing Value of Total Cholesterol in patients of OSMF**.

**Figure 3:**
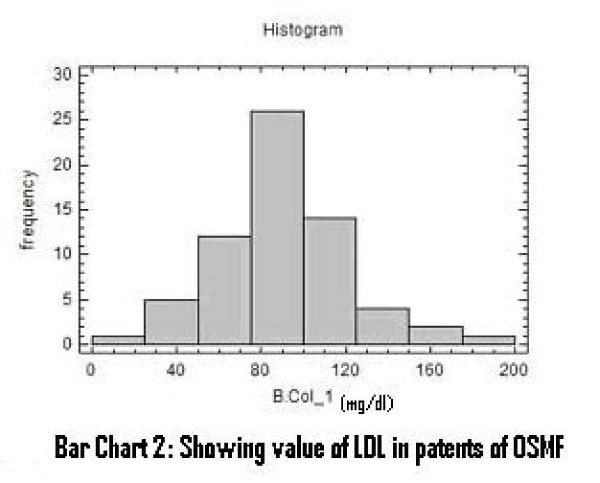
**Showing Value of LDL in patients of OSMF**.

**Figure 4:**
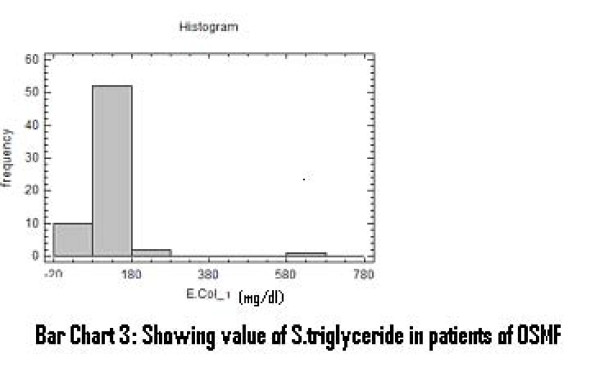
**Showing Value of S. triglyceride in patients of OSMF**.

**Figure 5:**
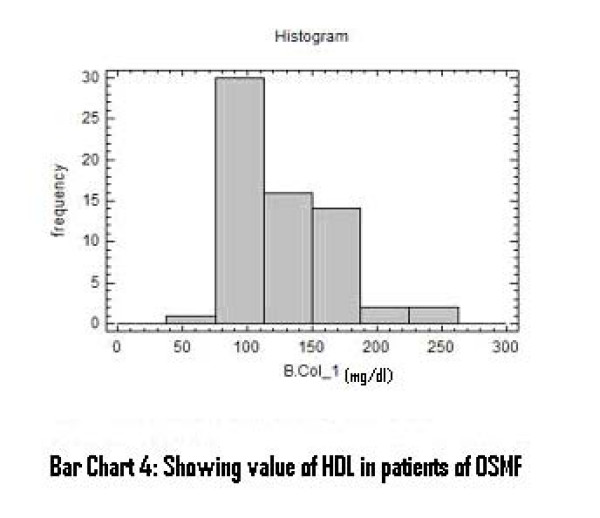
**Showing Value of HDL in patients of OSMF**.

**Figure 6:**
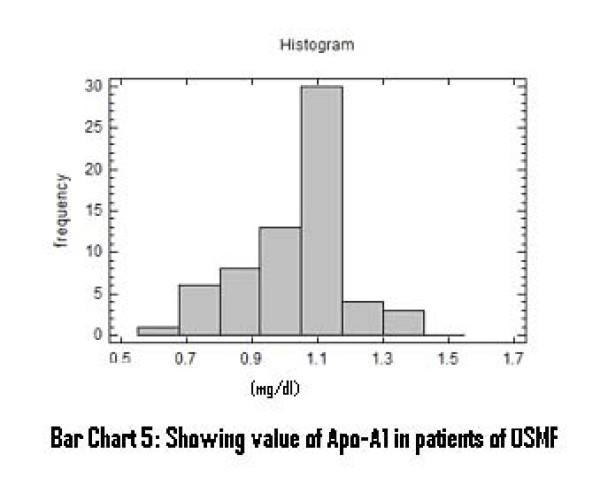
**Showing Value of Apo-A1 in patients of OSMF**.

**Figure 7:**
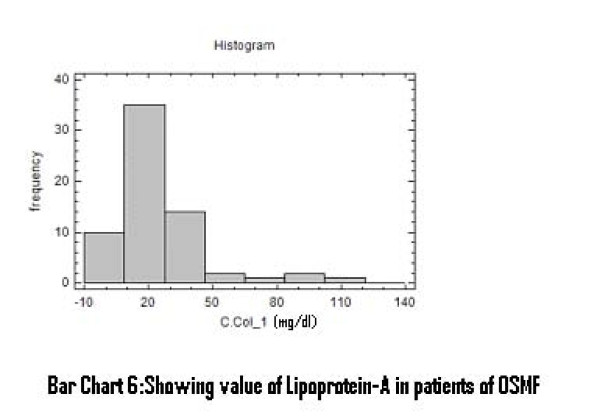
**Showing Value of Lipoprotein-A in patients of OSMF Bar**.

**Figure 8:**
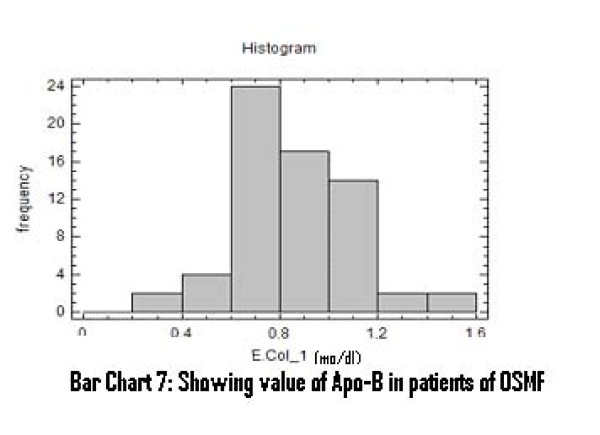
**Showing Value of Apo-B in patients of OSMF**.

There was a significant increase in serum total cholesterol, LDL, VLDL and triglycerides levels (Figure 2, 3, 4). On the other hand, there was significant decrease in HDL and Apo-A1 (Figure 5, 6). No change was observed in Apo-B or Lipoprotein-A (Figure 7, 8) in the patient category compared to the controls (Table 3).

**Table 3:** Value of Apo-A1, Apo-B, Cholesterol, HDL, LDL, Lp-A & S Triglyceride in OSMF Patients (Total N = 65)

	**Average** **Value** mg/dl	**Min-Max**	**Coefficient of** **Variation**	**Standard** **Deviation**	**Normal Value** **Min-Max**
Cholesterol	125.68	45.95–241.4	29.09%	37.42	130–250

HDL Cholesterol	32.58	15.36–70.4	30.83%	10.04	35–90

LDL Cholesterol	90.61	22.4–180.92	34.35%	31.13	0–129

S-Triglyceride	130.14	15.7–607	52.94%	68.90	60–165

Apo-A1	1.033	0.6–1.42	16.36%	0.169	1.20–1.76

Apo B	0.87	0.32–1.51	26.05%	0.227	0.63–1.76

Lipoprotein A	26.87	0.89–115.8	79.04%	21.24	< 30.0

## Discussion

Oral submucous fibrosis has always been a challenging disease with high prevalence in India. In this study, most of the OSMF cases were in the second and third decades of life with a male preponderance and addiction to areca nut. This is in accordance with previous reports from the authors as well other groups. As this is a disease of multifactorial etiology, various workers have proposed different theories of causation to establish the exact nature of the disease [11]. Pan Masala/Dohra were the most significant risk factor in this study but no correlation was found with the lipid profile Lipid Research Clinics Coronary Primary Prevention Trial was a multicentere, double blinded, placebo-controlled trial of the bile acid sequestrant, cholestyramine, among type Ha hypercholesterolemic men, i.e., men with elevated total serum cholesterol due to low-density lipoprotein cholesterol. The trial tested the hypothesis that long-term reduction of serum cholesterol leads to a lowered incidence of coronary heart disease. The results indicate that there is a decrease in plasma cholesterol levels associated with cancer apparent within 20 months of diagnosis in patients diagnosed with nonlocalized disease. Of the lipoprotein fractions, LDL-C most clearly reflects the decrease in total cholesterol.

The role of HDL-C and triglycerides in explaining the overall pattern of total cholesterol change is less clear [12].

Cholesterol is an amphipathic lipid and as such is an essential structural component of all cell membranes and of the outer layer of plasma lipoproteins. It is present in tissues and in plasma lipoprotein either as free cholesterol or combined with a long-chain fatty acid, as cholesteryl ester. It is synthesized in many tissues from acetyl-CoA and is ultimately eliminated from the body in the bile as cholesterol or bile salts [13]. Lipoprotein transports free cholesterol in the circulation, where it readily equilibrates cholesterol in other lipoproteins and in membranes.

Cholesteryl ester is a stored form of cholesterol found in most tissues. It is transported as cargo in the hydrophobic core of lipoproteins [14]. So, the fact that cholesterol in a hydrophobic molecule, which resides in lipoproteins and cell membranes, raises two questions: (i) how does the cell sense the level of cholesterol? (ii) how in this cholesterol specific signaling transmitted to the nucleus for the regulation of various genes? Recent studies directed to resolve these questions, led to the discovery of a novel cell surface cholesterol-sensor designated as receptor-Ck which was not only shown to be ubiquitously present in various human organs but also [through its signaling pathway] regulated various genes involved in cholesterol homeostasis [HMG CoA synthase; HMG CoA reductase; Apo-B-specific LDL – receptor]; cell growth [cyclin D; C – fos; C-myc; p27, etc.]; cell death [Bc1-2] through a 47 kDa transcription factor [derived from the cleavage of 125 kDa SREBP] having affinity for genomic sterol regulatory element [SRE] sequence as well as through other transcription factors [15-19].

Cholesterol is often found distributed non-randomly in domains in membranes [20]. Recent observations suggest that cholesterol exerts many of its actions by maintaining a specialized type of membrane domain, termed "lipid rafts" in a functional state. Lipid rafts are enriched in cholesterol and sphingolipids, and have been thought to act as platform through which signal transduction events are coordinated and pathogens gain entry to infect host cells [21].

In some malignancies, serum cholesterol undergoes early and significant changes. Low levels of cholesterol in the proliferating tissues and in blood compartments could be due to the rapidly dividing cells in malignancies. Several prospective and retrospective studies have shown an inverse association between blood lipid profiles and different cancers. [22]. Some scientists have observed an inverse trend between lower serum cholesterol and head and neck cancer as well as esophageal and colon cancers [9]. Similar findings were reported by Patel et al 2004 [10].

Earlier studies have shown alteration of plasma lipid profiles in head and neck and other cancers [10]. Lipids are important to carry out important biological functions. Cholesterol is vital for maintenance of integrity of biological membranes and cellular uptake. Regulation of cholesterol is mediated by lipoprotein receptors. Plasma triglycerides and cholesterol are packed into lipoproteins for transport. Cholesterol is an essential constituent of lipoprotein fractions like LDL, HDL and VLDL. Seventy five percent of the plasma cholesterol is transported in the form of LDL. Cells sequester cholesterol from LDL fraction of lipoproteins. Kesaniemi et al reported that LDL receptors are necessary for metabolizing circulating LDL levels and nearly 80% of the plasma LDL is cleared by LDL receptors [23]. High activity of LDL receptors attributes for lowering the serum cholesterol levels. The individuals having deficient or defective LDL receptors remove plasma LDL at much lower rate and have considerably elevated levels [24].

HDL and Apo-A_1 _levles may be a useful indicator reflecting initial changes occurring in pre-cancerous and neoplastic conditions. A significant decrease in levels of HDL and Apo-A1 was also observed in this study. This was in accordance with previous reports [10,25] which reported that lower HDL is an additional predictor of oral pre-malignant condition and it might be a consequence of disease that is mediated by utilization of cholesterol by membrane biogenesis. Schatzkin et al also observed an inverse relationship between serum cholesterol and oral pre-malignant condition [26].

### Future strategies

The evidence suggests that precancerous lesions are able to remodel/metabolize lipids for their growth and to generate phospholipids membrane. Thus, the identification and characterization of more enzymes involved in these pathways will be more important for the proper understanding of these steps. Recent progress in molecular biology will assist researchers in the near future to identify the genes and enzymes of lipid metabolic pathways.

The change in lipid levels may have a diagnostic or prognostic role in the early diagnosis or prognostication of oral premalignant and malignant lesions. The findings strongly warrant an in-depth study of alterations in plasma lipid profile in these patients.

## Abbreviations

HDL: High Density Lipoprotein; LDL: Low density lipoprotein; VLDL: Very Low Density Lipoprotein; SRE: sterol regulatory element; LPa: Lipoprotein A; OSMF: Oral submucous fibrosis.

## Competing interests

The authors declare that they have no competing interests.

## Authors' contributions

SP and AKC drafted and analysis the manuscript. HPS and RKS carried out the experimental work. RM and SCG conceived of the study, participated in its design and coordination as well as helped to draft the manuscript. MS and Mamta Singh participated in coordination of the study and helped to draft the manuscript. All authors read and approved the final manuscript.
